# Her Heart's Mistake

**Published:** 1889-09-07

**Authors:** E. D. Cumming

**Affiliations:** Author of "An Ordeal by Silence"


### Her Heart's Mistake.

By E. D. Cumming, Author of "An Ordeal by Silence."

*(Continued from page 332.)*

### Chapter XXI.—"For Better or for Worse."

Warren read the paper as a man might read his death-warrant. He returned it to the Doctor, and for a few moments they stood facing one another in silence.

"Had I seen the message come to the house I would have taken upon myself to suppress it," said Dr. Harrison. "It has thrown her into a worse state than ever, and I cannot even get her to speak."

"May I see her?" asked Warren, striving to speak calmly.

"It is cruel to say no, my dear fellow, but you will believe me when I tell you that I am now seriously alarmed about her. Her mind will give way if this goes on much longer."

"What are we to do, Doctor?"

"God help me, Warren, I don't know. I will not risk agitating her now by mentioning that blackguard's name. We must not attempt to argue with her at present, and the sight of you would only increase the mental strain."

Warren folded his arms, and stared blindly out into the compound. The cup had been dashed from his lips almost as it touched them. He felt that if once Bairdsley should see Kate she would be hopelessly lost to him.

"I shall remain here all day in order to prevent his coming near her," said the Doctor, crumpling the fateful telegram in his hand. "I shall try what I can do to impress him with a proper sense of his conduct before I allow Kate to see him. I don't believe she is equal to the task of making another bid for freedom in the state she is now."

And truly the girl, crouching on the floor of her bedroom amid a litter of half-filled trunks, did not look as though she belonged to the world of the living at all. She sat motionless, with her head bent forward, as if listening for an approaching footstep. Her eyes were fixed on vacancy, and her hands had lain idly in her lap since they had opened Bairdsley's telegram. She had sent it out to her father, and had neither moved nor spoken since.

He came in to her again as soon as Warren had gone, and, lifting her in his arms, laid her upon the bed. She made no resistance, seeming hardly conscious of his presence, and ate and drank mechanically when he fed her. He did not go up to the hospital that morning; he remained at his daughter's side, but without making any attempt to rouse her. He felt that he must pin his hopes to Bairdsley's good feeling, and could not believe that any man would persevere in a line of action which had produced such results as this.

At half-past one he rose from the seat he had occupied for nearly four hours, and left the room, carefully closing the doors, that Bairdsley's approach might not be heard within. He wished now that he had gone up to the railway station to intercept him there, but his thoughts had been so full of his daughter that the step had not suggested itself. He walked up and down the verandah with a beating heart; he must be cool, he told himself over and over again; he must not allow his anger to get the upper hand of him whatever the man might say or do.

Bairdsley appeared at length, on foot. He had left his baggage at Captain Phelps's bungalow, and had come straight over to the Doctor's. He entered the verandah, and advanced with outstretched hand, with as much nonchalance as though nothing of an unpleasant nature had taken place since he left Meerut. He was thinner than he had been upon his first arrival in the country, but otherwise looked healthy and well.

"Where is Kate?" he enquired. "I asked her to come and meet me."

The man's selfishness was only equalled by his effrontery, and it cost the Doctor a mighty effort to preserve his tranquil bearing. He did not notice the hand held out to him; he led the way to the far end of the verandah, and beckoned him to follow.

"My daughter is too unwell to see you," he said, "and even were she able to do so, I should not permit it."

Bairdsley laughed harshly, but checked himself at once. "I'm sorry to hear she is seedy; I hope she has got over that absurd idea about breaking off her engagement to me. It gave me a great deal of pain, I assure you."

He was determined to maintain the farce of believing that she loved him; and the Doctor forced himself to come to the point at once.

"I must ask you to give up your claim," he said. "I will not dwell upon the astonishment with which I have read your letters to her, or that which you addressed to me. It is impossible that you can wish to marry her after what she has written you; I could not credit the evidence of my senses when your last communication reached her."

Bairdsley turned red with anger, and the sight of his wrath had the natural effect of steadying the Doctor.

"I claim fulfilment of the promise she made me," he said. "If she tells me flatly that she won't keep it, why, of course, I can't marry her by force, but I will have my dismissal from her own lips. I wish to see her at once."

"I positively refuse to allow it," said Dr. Harrison with decision. "Your conduct is affecting her most gravely as it is. I dare not think what the consequences may be if you persevere in the course you have adopted."

"In what respect, pray?"

"Upon her reason, sir! Upon her mind! You know how she is placed. She made a girl's mistake, and has a mistaken sense of honour, and of both you are taking the most despicable advantage. I cannot trust myself to speak plainly to you. Have the goodness to leave my house, and ask my permission before you enter it again."

The Doctor pointed to the compound gate with a hand that shook with passion. Bairdsley had received his speech about poor Kate's mistake with an insolent smile that drove him mad with rage, and he could not restrain himself longer. His visitor drew himself up and hesitated for an instant, then shrugged his shoulders and turned upon his heel.

"I shall ask your leave to return when Kate is able to see me," he said as he went out. Dr. Harrison made no reply, but watched him leave the premises, and returned to his daughter. She was sitting up and her face was flushed with excitement.

"He is here," she said quickly, as her father entered the room.

"He was here, but he has gone away again and won't come back till he is sent for," answered the Doctor,

"I must see him," said Kate, who had been completely roused from her apathy by Bairdsley's voice. "Send for him at once, father; please send after him and tell him I want to see him now."

"Not just yet, dear. If you are well enough to see him this evening, you shall."

Kate thought for a minute, and acquiesced in the suggestion.

"What did he say about it?" she asked almost indifferently.

"He said that he would take his dismissal only from you in person," replied her father. "I knew that it was impossible that he would persist in this outrageous behaviour. All you have to do now is to bid him begone. Now lie down and go to sleep for a while."

Kate lay back on her pillows with a long-drawn sigh; she had come to the end of her struggle at last.

"You will send over, and tell him to come this evening?" she said, as Dr. Harrison drew the purdahs, to exclude the light. "Promise to send for him this evening."

"I'll tell him to be here at six, if that will suit you."

Kate only nodded in answer, and as though a crushing weight had fallen from her, closed her eyes, and fell asleep. It was nearly dark when she awakened, and her ayah, who had been on the watch for her to move, came in with the sympathetic smile of one who brings good news.

"Captan Bairdslee sahib, waiting, waiting," she said, pointing to the verandah.

Kate rose, and hastily prepared herself to go out and receive him. She was trembling in every limb, but sustained herself by recollecting that this was the last page of the story. He shook hands as he might have done with an ordinary acquaintance, and sat down on the lounge beside her.

Till the last day of her life that interview remained the darkest spot in Kate Harrison's memory. She met Bairdsley prepared to receive her release; she left him having renewed her pledge to become his wife. The strength of his personality overawed her at the outset, and gradually she felt the power of thinking for herself slip from her, and her very individuality melt away before the heat of his passion. Until long afterwards she never recognised the skill with which he played upon her morbidly conscientious nature. She knew that ere he had been ten minutes at her side, tender reproachful words, such as the man she had once believed him to be, might have spoken, had revived in tenfold strength her first remorse. She was the sinner, he the wronged. She was the callous, selfish woman, he the patient, forgiving sufferer. She saw her disloyalty and fickleness with his eyes. She realised the justice of his anger, and the cruelty of her defection. His vehemence left her no leisure to think of anything but the reparation due to him, and the stern necessity for paying the full penalty of her mistake. He showed her the strength of her chains, and, true to his falsity, bade her break them if she would; he had come to receive his dismissal if she wished to give it.

"Her behaviour is perfectly inexplicable," said Mrs. Tenterdale, a few evenings afterwards, to a select party of friends who had dropped to in tea. "Captain Bairdsley has treated her most indulgently. Very few men have been so forgiving."

"From whom did you hear about it, Mrs. Tenterdale?" enquired Mrs. Arkwright. "I went to see Kate to-day, but could learn nothing except that the wedding was fixed for Friday. She would hardly speak about it.'

"Captain Bairdsley told me all about it. She wrote him half-a-dozen times begging to have the engagement off; and at last he grew alarmed and came down. As soon as she saw him she turned round again and implored him not to give her up. It's extraordinary."

"That man Warren was at the bottom of it, I believe," said Mrs. Strachan; "at least everyone appears to think so."

"Well, it's all settled now. They are to be married on Friday afternoon at half-past four."

"Dr. Harrison has been looking very serious lately. I spoke to him about Kate yesterday, and he looked as black as a thundercloud," said Mrs. Arkwright; "I was positively frightened."

"Her conduct has annoyed him very much; and no wonder," said Mrs. Tenterdaie. "What are you going to wear on Friday?"

The conversation drifted into the labryinth of dress, and lost itself there. Nothing had been known about Miss Harrison's attempt to sever her engagement with Captain Bairdsley until his sudden return from the hills attracted remark; and he, recognising that the station was in ignorance on the subject, had given a plain, straightforward account of it to his Colonel's wife. The Captain disliked scandal, particularly when his own concerns were involved, and was devoutly glad that he had come upon the scene in time to nip any chatter in the bud. He knew that the matter could not be kept secret, and wisely forestalled garbled stories by giving out his own.

Meerut Cantonment Church was full that Friday afternoon; every pew was occupied by ladies in their gayest attire, and men in the brilliant uniforms of English and native cavalry and infantry. Red tunics and blue, which had not seen the light of day since last cold weather, appeared for once to honour the occasion. No Karkee or white for a wedding! Full dress was the order of the day, and the aisles were a mass of colour save for the sprinkling of black-coated civilians.

George Bairdsley was cool and composed; but his bride's demeanour was even more collected, and excited general attention.

"She looks as though she were walking in her sleep," said one lady to her neighbour, in an awestruck whisper.

"Might be a marble statue," returned the other.

She moved steadily, but her face was devoid of all expression from the moment she entered the church until the clergyman closed his book. She was in a dream, and hardly knew when the ceremony was over.

"Did you see Mr. Warren in church?" asked Mrs. Arkwright eagerly of Captain Andrews, as the congregation poured out of the doors.

"No, I don't think he was there."

He was not there. He was sitting on the bed in his room at the club, with his hands tightly clenched together, and a look of set purpose on his ashy face. He had not seen Kate nor heard a word from her since that memorable night a week ago, when she consented to marry him. Many tongues had told him what had happened since, and all had the same tale, Bairdsley's tale, to tell. He sat there, thinking, brooding over the wreck of his life, until the repeated rumble of carriage wheels told him that the wedding party was returning from church to the corner bungalow. Presently he pulled himself together and looked round the dark room. All was silent again without; the only sound he could hear now was an occasional cry from a native servant, or the stamp of a horse in the stables behind. The yellow lizards "chuck-chucked" on the wall as they chased the flies, and noticed what the man in the room was doing as little as he heeded them. He rose slowly from his seat on the bed, and drew near the dressing-table with a step of a frightened thief. He rested both hands upon it for a moment, and a terrible shiver ran through his frame as he stood. He took a razor from the case, and, opening it, laid it down and fixed his eyes upon the bright steel until they almost started from his head, and the perspiration broke out and fell in heavy drops from his brow. He raised the weapon in his right hand, leaning upon the table with the other. His eyes were tightly shut now, and he had the razor firmly between his fingers as he whispered to himself that another moment would bring him his release. He opened his eyes and tried the edge upon his bare arm until it was dyed red and the blood ran down upon the floor. The sight seemed to bring him to himself. He stood upright, and, with an effort of will, flung the razor down. But he could not trust himself to remain in the room longer, and he rushed out, hatless and coatless, into the open air. Whither his feet took him he did not care; they could not help him to escape from himself, go where they would; he was, for the time, mad.

(*To be continued.*)

